# Reviews of Books

**Published:** 1924-09

**Authors:** 


### REVIEWS OF BOOKS.

### THE DEVELOPMENT OF MEDICINE.

**Hippocrates and his Successors in Relation to the Philosophy of their Time.** Fitzpatrick Lectures, 1921-22. By R. O. MOON, M.D., F.R.C.P. (Longman. 6s. net.)

**The History of Physiology during the XVIth, XVIIth and XVIIIth Centuries.** By Sir MICHAEL FOSTER, M.D., F.R.S. (Camb. Univ. Press. 15s. net.)

No finer plea for knowledge of the historical development of science could be found than that given by Sir Michael Foster in the opening words of his lectures, now reprinted, given in San Francisco a quarter of a century ago :—

What we are is in part only of our own making. The greater part of ourselves has come down from the past. What we know and what we think is not a new fountain gushing forth from the barren rock of the unknown at the stroke of the rod of our own intellect, it is a stream which flows by us and through us, fed by the far-off rivulets of long ago. . . . Tracking out how new thoughts are linked to old ones, seeing how an error cast into the stream of knowledge leaves a streak lasting through many changes of the ways of man . . . we may perhaps the better learn to appraise our present knowledge, and the more rightly judge which of the thoughts of to-day is on the direct line of progress, carrying the truth of yesterday on to that of to-morrow, and which is a mere fragment of the hour, floating conspicuous on the surface now, but destined soon to sink, and later to be wholly forgot.

The study of history plays the same part in medicine as the spectroscope in astronomy, for it enables the student to understand the relative movements of the ideas and theories which enter into our present knowledge. Without the power to turn our thoughts back along the path towards a far-receding past we cannot rightly envisage the true relationships of the knowledge of to-day and are but mean artificers without a complete grasp of our craft. In the domain of thought there is a determinism as strict as that which exists in the physical world, and each advance rests on the foundations already laid by previous thinkers. Though Hippocrates, like Sydenham in his turn, was hailed as "the father of medicine," he owes a heavy debt to his predecessors. What this indebtedness is, Dr. Moon has essayed to show in his Fitzpatrick Lectures for 1921-22.

Hippocrates was first and foremost a practising physician who "made medicine a science distinct from all others, having its own principles and methods of exposition, and seeking in itself its principles of development. But he did not trumpet forth an exclusive system, or a new doctrine, for indeed he drew most of the elements of his science from tradition." But the versatile enquiring mind of the Greek was above all things free, and was never content to accept the *ipse dixit* of the master in place of investigation, and the authority of Hippocrates never laid its dead hand upon speculative thought as that of Galen did in after years. Hence, under the influence of the great schools of philosophy there sprang into existence one school of medical thought after another—the Dogmatists, the Empirics, the Methodists and the Pneumatists. It is in the detailed exposition of the teaching and tenets of these schools and the relationships of their founders to current philosophical speculation that Dr. Moon has given us most valuable aid. It is a source of gratification that, in what is fast becoming a "Greekless age," there are still to be found in the ranks of medicine scholars of the type of Dr. Moon who worthily uphold the reputation for learning and scholarship which has always been associated with the Royal College of Physicians.

The subject matter of Sir Michael Foster's lectures offers a most interesting contrast to that of Dr. Moon, for they describe the desperate struggle for independence of thought and freedom of expression, which for more than a millennium had been fettered by the shackles of intellectual authority aided by the material power of an ignorant and grievously mistaken Church. Vesalius, who, harassed by the minions of the Inquisition, was prevented from a full and complete exposition of his views ; Servetus, who suffered at the stake for his opinions ; Borelli and Galileo, who barely escaped the same fate, all afford evidence of the bitter opposition with which the search after Truth was assailed. But *magna est veritas et praevalebit*, and the difficulties of the protagonists in the struggle opened the way to a fuller realisation that knowledge cannot be confined within the narrow walls of arid dogma. Slowly but surely the battle was won. Sir Michael has made each actor in the story he unfolds speak for himself by giving extracts from his writings. These lectures are not, however, mere compilations, for they are expositions with that lucidity of style and grasp of his subject which bespeak the master. To those whose privilege it was to listen to Sir Michael Foster's lectures at Cambridge, and to be introduced to the study of physiology by his well-known textbook, these lectures will bring back memories of that grave dignity of presence, those little personal traits and turns of phrase which made his lectures a constant pleasure. The Cambridge University Press deserves our gratitude for the republication of these lectures, which have a permanent historical value.

### THE WHY OF MURDER.

**Murder and its Motives.** By F. TENNYSON JESSE. (Heinemann. 8s. 6d. net.)

Books about crime, and especially about murder, are always attractive, whether the motive of the reader be curiosity, psychology or the mere love of an exciting story. Miss Jesse's book is certain, therefore, to be popular, notwithstanding the pseudo-scientific idea which lies behind it. That idea is the division of murder into categories—murder for gain, for revenge, for jealousy, for sheer love of killing, for conviction, for desire to eliminate a person who is in the murderer's way. The one crime for "conviction" with which she deals is that of Orsini ; the murder of "conviction," consequently, means mere political crime, such as that of which Nihilists and Bolshevists and other political assassins have so often been guilty. In her preliminary chapter on "The Classification of Motives" Miss Jesse writes with a certain air of philosophic profundity, but what she has to say is all rather obvious. We do not as a rule need a philosopher to tell us the reason for a particular murder—once find your murderer and it is usually easy enough to discover his motive. Nor has Miss Jesse much that is new or suggestive to say about murderers and their motives. It is, indeed, doubtful whether anybody would be able to say anything new on the subject, or whether even the most profound philosopher could get far above high-class journalism in his comments upon the why and the wherefore of the worst of crimes.

As a story-teller Miss Jesse is in another category. That is her true business. Her narratives are excellent, especially when they are not clogged by reflections. Her subjects are well chosen for her purpose, though only one of them, that of the De Quérangals, presents any novelty to English readers. She tells the tale of the numberless poisonings of Palmer of Rugeley with dramatic simplicity and full command of the facts, though reference to a gazetteer would have told her that he could not have been apprenticed to a surgeon at "Hayward" because there is no place of that name in this country. The place was Great Haywood, very near to Rugeley, where Palmer lived and poisoned. He was the murderer for gain. The tragedy of Constance Kent is also told clearly and well, though why Miss Jesse should censure Mr. Kent for having children by his second wife is not obvious. Then we have the familiar stories of Mrs. Pearcey (jealousy) and Neill Cream (the lust of killing). The De Quérangals' crimes were for elimination, and they make a strange, if sordid, story. Orsini, the murderer "for conviction," pretended to be a patriot, but he was a mere wholesale killer who cared not how many he killed so long as Napoleon III. was among them. Miss Jesse thinks that there was "something about the man that inspires respect." We do not; he killed fourteen people, but missed the man at whom he aimed. And surely she stultifies herself when, after writing the words we have quoted, she goes on to say that Orsini was "a worse criminal from the point of view of the community than the ordinary criminal." That is the true verdict upon all fanatics who kill for political reasons—reasons which the common-sense law of England refuses to recognise.

New generations are constantly growing up to whom even such oft-told tales as those of Palmer and Constance Kent are fresh, and these new readers will find Miss Jesse's narratives full of gruesome interest. But she has hardly done more than add another volume to the enormous literature of criminal trials.

### THE SPIRIT OF JOHN HUNTER.

**Orations and Addresses.** By Sir John Bland-Sutton. (Heinemann. 10s. 6d. net.)

General literature owes much to members of the medical profession. Nor has there ever been lacking a supply of surgeons able to present, in acceptable literary form, the things which appertain to their science. Sir John Bland-Sutton is an eminent example. In this volume he has collected and republished a number of addresses delivered on various occasions and in divers places. The subjects range from the historical eulogy pronounced on John Hunter at the Royal College of Surgeons last year, to cranial morphology and choroid plexuses. It is needless to make any attempt to summarise these addresses; they can all be read with profit and admiration.

But a message runs throughout the book. The scientific spirit of Hunter should be the inspiration of surgeons to-day. Although active theorising is essential for progress in any branch of learning, instances are given of the care with which Hunter verified his experiments by careful "controls." Sir John Bland-Sutton well says that surgeons are of two kinds—they are either craftsmen or biologists. True craftsmanship is, of course, essential, but the scientific spirit is even more so. And the true path to surgical success runs by way of the biological laboratory and the pathological institute. Many of the foes of daring surgeons of the past were found among those of their own household. We are reminded how the first microscopists were laughed at as "brass and glass men." Even to-day the "spirit of the profession remains unchanged." Much of the opposition to recent surgical discoveries has come from those who are content to perpetuate the errors and to practise the ritual of their predecessors. The author points this golden moral—that it is, before all things, necessary that true surgeons should remember that they are not competitors but comrades of the same honoured craft or guild.

The book is splendidly printed and most handsomely illustrated. We can imagine no better gift for a surgeon or medical student.

### ANDROLOGY.

**Diseases of the Male Organs of Generation.** By K. M. Walker, O.B.E., F.R.C.S. (Oxford Medical Publications. 12s. 6d. net.)

The author has set himself the task of presenting to general practitioners and students the main features of the various morbid conditions of the generative organs of the male. Seeing the important, if obscure, part played by the prostate in the generative functions, it is not surprising to find that affections of this gland and of the seminal vesicles occupy nearly one-third of the volume. As the author remarks, the only certain cause of enlargement of the prostate is old age, but, as a result of Mr. Walker's investigations, no explanation can be found to account for the rarity of the condition among certain races. If Mongolians are free from the trouble, the white and the Semetic races appear to be the most affected. Much sound advice is given as to the management of prostatics in general, for much may be done in the stage of irritability in the way of relieving congestion which may postpone operation. Nevertheless, it is pointed out that if definite symptoms of obstruction have appeared, and there is an increasing amount of residual urine in the bladder, the question of operative interference must be seriously faced. The two-stage operation is preferred by the author, a preliminary suprapubic cystotomy being first performed, followed at a later period by enucleation.

With regard to genital tuberculosis in the male, the author believes that the clinical and pathological evidence points to the fact that the disease generally spreads "as a descending wave from a primary focus in the prostate or vesicles" by way of the lymphatics surrounding the vas. The results of orchidectomy have proved somewhat disappointing, and unless it be desired to rid the patient of a badly disorganised organ it should seldom be performed. It would appear to be more logical to employ the radical method advocated by Hampton Young and to excise the whole seminal tract. Although this is a more severe operation, it is claimed that its results are superior to those obtained by other surgical procedures. The use of tuberculin is recommended in certain cases, but the treatment by this means is necessarily prolonged.

During recent years considerable attention has been focused upon the part played by the interstitial cells and upon the internal secretion of the testis. The "rejuvenation" experiments of Steinach have excited great interest among medical men and scientists, and even the general public has now some inkling of the use to which "monkey glands" are being put. The author adopts an attitude of reserve towards the whole subject, and considers that it must still remain *sub judice*. Chapters upon male sterility and functional sexual disorders bring to a close a volume that fulfils the highest traditions of the series of which it is a most worthy number.

### YEARS AND HONOURS.

**A Long Life's Work: An Autobiography.** By Sir Archibald Geikie, O.M., K.C.B. (Macmillan. 18s. net.)

When a worker in a single field of knowledge has watched, through a long span of years, his subject grow from infancy to a full maturity, there seems to be almost a reversal of the truth of the ancient adage which contrasts the shortness of human life with the durability of science. Sir Archibald Geikie, now in his eighty-ninth year, has seen the science of geology develop in just such a way, and in that development has himself played no inconsiderable a part. We are sometimes tempted to ask: "Where are the giants of the past?" forgetful that, as knowledge widens, it is the small additions to the sum total which tell, here a little and there a little, rather than the enunciation of new principles and sudden great advances which confer immortality upon their discoverers. It is only in the infancy of a science that the names of such men as George Scrope, Sir Charles Lyell, Sir Roderick Murchison, Sir H. de la Beche and Hugh Miller can stand out so clearly above their fellows. It is the fact that Sir Archibald Geikie is a still-living connecting link with all these pioneers which gives so special an interest to his autobiography.

Born into a cultured home in Edinburgh—his father was for many years musical critic to the *Scotsman*—Sir Archibald received that most valuable of all possessions, a sound classical education, which has afforded him a lifelong pleasure and has given to his numerous books a distinction and style too often lacking in scientific treatises. Without private means which might have enabled him to pursue the calling of his choice, he was constrained to enter the office of a Writer to the Signet as a preliminary to a career in a bank. Probably this constraint was a blessing in disguise, which proved its value in after years when he became Director of the Geological Survey, subsequently Secretary, and still later President of the Royal Society. To this humdrum discipline of early life he owes another debt, best expressed in the words of his own favourite Horace:—

> Qui cupit optatam cursu contingere metam,
> Multa tulit fecitque puer, sudavit et alsit.

No doubt Fortune played her part as Fairy Godmother, for it was his luck to meet with various persons who were able to use influence in the right quarters and at the opportune time, but the ground had already been well prepared by the lad's keenness to learn all he could. The find of a fossil plant which he could not identify brought him to the notice of Hugh Miller, who recommended him to Murchison, when he was about to extend the Geological Survey to Scotland. Geikie's innate capacity and application to his work did the rest and carried him rung by rung to the top of the ladder. His life has been comparatively uneventful, yet academic and other honours came to him thick and fast, crowned at last by the highest that British Science has to bestow—the Presidency of the Royal Society. Of his labours the progress of the Geological Survey, and the memoirs published in connection therewith, form an enduring monument, while his *Ancient Volcanoes of Great Britain* will remain a mine of information.

### MISCELLANEOUS NOTICES.

### Directory of Medical and Nursing Homes.

Medical and Nursing Homes, 1924-1925. (Scientific Press. 9d.)—This directory has been compiled for the convenience of the medical profession and public seeking information in regard to sanatoria and trustworthy Homes of all descriptions. It also gives a list of schools for delicate children. It is cross-indexed, is of a convenient pocket size, and should prove very useful.

### Dental Anæsthetics.

Anæsthesia in Dental Surgery. By T. D. Luke, M.D., and I. Stewart Ross, M.B. (Heinemann 10s. 6d. net.)—Recent improvements in anæsthetic administration, particularly in dental work, make this fifth edition doubly welcome. The book retains its essential character as a guide to the practitioner to whose lot it frequently falls to give dental anæsthetics. Particular attention is paid to gas and oxygen, and mention is made of all modern methods and apparatus. Another useful section is that dealing with the pharmacological action of the various drugs used in conjunction with direct anæsthetics.

### For the Practising Physician.

Organic Substances, Sera and Vaccines. By D. W. Carmat-Jones, M.D. (Heinemann. 15s. net.)—This book of nearly 400 pages bears the stamp of scientific honesty: the author's aim has obviously been to furnish the profession with a reasoned exposition of his subject. His facts are taken from his own large experience and from the work of others; they are marshalled in orderly sequence and their significance is discussed with a fairness of judgment which disarms criticism. No longer can the subjects with which he deals be dismissed as fads and fancies from which sound medicine should be free, but neither should ill-balanced enthusiasm be allowed to assign them too exalted a position in the realm of treatment. It is here that the considered opinion of a sound physician is so valuable. Practice has not been subordinated to theory, the practical details of dosage are given and the indications and contra-indications for the various methods are discussed. This book is an indispensable addition to the library of the practising physician; it is well written, accurate in its statements and very readable. Author and publisher are to be congratulated upon their work: further editions will certainly be demanded.

